# Validation and Diagnostic Efficiency of the Mini-SPIN in Spanish-Speaking Adolescents

**DOI:** 10.1371/journal.pone.0135862

**Published:** 2015-08-28

**Authors:** LuisJoaquín Garcia-Lopez, Harry T. A. Moore

**Affiliations:** 1 University of Jaen, Jaen, Spain; 2 University of Amsterdam, Amsterdam, The Netherlands; University of New South Wales, AUSTRALIA

## Abstract

**Objectives:**

Social Anxiety Disorder (SAD) is one of the most common mental disorders in adolescence. Many validated psychometric tools are available to diagnose individuals with SAD efficaciously. However, there is a demand for shortened self-report instruments that identify adolescents at risk of developing SAD. We validate the Mini-SPIN and its diagnostic efficiency in overcoming this problem in Spanish-speaking adolescents in Spain.

**Methods:**

The psychometric properties of the 3-item Mini-SPIN scale for adolescents were assessed in a community (study 1) and clinical sample (study 2).

**Results:**

Study 1 consisted of 573 adolescents, and found the Mini-SPIN to have appropriate internal consistency and high construct validity. Study 2 consisted of 354 adolescents (147 participants diagnosed with SAD and 207 healthy controls). Data revealed that the Mini-SPIN has good internal consistency, high construct validity and adequate diagnostic efficiency.

**Conclusions:**

Our findings suggest that the Mini-SPIN has good psychometric properties on clinical and healthy control adolescents and general population, which indicates that it can be used as a screening tool in Spanish-speaking adolescents. Cut-off scores are provided.

## Introduction

Social anxiety disorder (SAD) tends to be a chronic condition that severely disrupts long-term functioning [1]. Epidemiological and clinical samples have revealed that SAD usually develops in adolescence and is stable into adulthood [2]. The estimated mean lifetime prevalence in adolescents ranges between 2 and 9%, which makes SAD one of the most common mental disorders in adolescence [3].

Significant progress in the assessment of adolescents with SAD has been made over the last decades. More adolescents with SAD are being identified and accurately diagnosed and a number of psychometrically sound assessment measures are available [4]. However, many questions remain to be answered about how to efficiently detect adolescent populations who are suffering from or at risk of suffering from SAD. Early risk screening requires sensitive instruments to identify adolescents at risk of developing SAD and who are in need of comprehensive assessment and treatment. The gold-standard instrument for screening adolescents with SAD should be brief, reliable with high sensitivity and specificity, able to identify youths for further clinical assessment and treatment interventions, easy to administer and cost-effective [5]. However, only a few social anxiety screening measures for adolescents are available. In addition, a measure’s psychometric properties must be tested in different cultural groups and contexts.

The Mini-Social Phobia Inventory (Mini-SPIN) [6] is a brief 3-item scale derived from the SPIN and has recently received increasing attention for its potential use as a screening measure. The Mini-SPIN has been evaluated across different cultures, languages and age groups. In regard to adult English-speaking samples, Connor et al. [6] found the Mini-SPIN to have optimal diagnostic efficiency at a cutpoint of 6 or greater with a sensitivity of 89% and specificity of 90%, while Seeley-Wait et al. [7] also found optimal diagnostic efficiency at 6 or greater with a sensitivity of 88% and specificity of 98%. Similarly, the Mini-SPIN has also been found in other English-speaking samples to have a good sensitivity of 94% at a cutpoint of 6 or greater, although and a relatively lower specificity of 64% [8]. The Mini-SPIN has also been evaluated in a Brazilian university sample (mean age = 21) with optimal diagnostic efficiency at 7 or greater with a sensitivity of 78% and specificity of 68%. [9]. The only evaluation of the Mini-SPIN on adolescents (between 12 and 17 years) comes from a Finnish-speaking sample which found optimal diagnostic efficiency at 6 or greater with a sensitivity of 86% and specificity of 84% [10]. The Mini-SPIN has also been found to have good discriminative validity between mental health disorders [7, 8, 10] and is sensible to treatment change [7].

The present study aimed to determine the psychometric properties of the Mini-SPIN in order to screen for adolescents in another culture and language with and without SAD from the wider community (study 1), and clinical and healthy control samples after diagnostic assessment (study 2). Our target population was Spanish-speaking adolescents in Spain.

## Study 1

### 2. Method

#### 2.1. Participants

The community sample consisted of 573 participants aged between 12 and 18 years old (M = 15.04, SD = 1.33), of which 267 (46.6%) were boys and 306 (53.4%) were girls. Participants were recruited from 1 private and 2 public high schools in a medium-size state in the south of Spain. Schools were selected by a clustered random sampling method from the school lists of the Department of Education to ensure socioeconomic status and ethnic compositions of the sample were representative of the community.

#### 2.2. Measures

Mini Social Phobia Inventory (Mini*-SPIN)*. The Mini-SPIN [6] consists of three items taken from the Spanish translation of the Social Phobia Inventory (SPIN; [11]). The original SPIN [12] consists of 17 items that measure fear, avoidance and physiological discomfort in social situations. The Mini-SPIN contains items 6, 9 and 15 from the original SPIN. Items are rated on a 5-point Likert scale (1 = not at all, 5 = extremely). The Mini-SPIN has been found to be a valid and reliable measure of SAD for screening socially anxious adolescents in Finland [10], as well as in adult populations in English-speaking countries [6–8]. The Mini-SPIN is available for free use, and have no cost associated with scoring.

Social Phobia and Anxiety Inventory-Brief (SPAI-B). The SPAI-B [13] is a brief version of the 45-item SPAI [14]. The SPAI-B measures cognitive, behavioural and somatic symptoms of social anxiety. Unlike the SPAI, the SPAI-B presents items with a different likert scale format and fewer items, and does not use heterocentric language [15]. Sixteen items are rated on a 5-point likert scale (1 = never, 5 = always). This scale has been validated for adolescents and young adults in both online and paper-and-pencil format, and particularly for use as a screening measure, with a rounded cut-off point of 26 [16, 17, 18].

Social Anxiety Scale for Adolescents (SAS-A). The SAS-A is an adaptation of the Social Anxiety Scale for Children-Revised (SASC-R) for adolescent populations [19]. The SAS-A consists of 22 items presented on a 5 point Likert scale (1 = never, 5 = always). Eighteen items are descriptive self-statements and four items are filler items. The SAS-A contains three subscales: Fear of Negative Evaluation (FNE: 8 items), Social Avoidance and Distress specific to new situations or unfamiliar peers (SAD-N; 6 items), and Social Avoidance and Distress that is experienced more generally in the company of peers (SAD-G; 4 items). Studies conducted in western and eastern cultures have reported excellent psychometric properties for this measure of social anxiety in adolescents (for a review, please see [20]).

#### 2.3. Procedure

Prior to the study, the participants and their parents were informed about the study and its goals. Active informed consent was obtained from the adolescents’ parents before the research was conducted. In addition, students whose parents or legal guardian signed the consent forms and returned it by the assessment date participated in the study (parent consent rate: 68%). The adolescents provided assent before the study and could at any time decline to participate in the study. Students completed the self-report inventory in their classrooms. Completion of the scales took approximately 40 minutes. Ten research assistants received background information on the assessment of social anxiety, and administering the Mini-SPIN, SAS-A and SPAI-B in particular, in school settings during two 45-minute sessions. The study was approved by the Management Committee in each high school and the University Research Ethics Board as IRB, in compliance with the Code of Ethics of the World Medical Association (Declaration of Helsinki) and the Charter of Fundamental Rights of the European Union.

#### 2.4. Statistical analysis

Analyses of variance (ANOVA) were conducted for the sum score of the Mini-SPIN, with age and gender as between-subject factors. Cronbach’s alpha, greatest lower bound (glb) and the omega total coefficient examined internal consistency. The alpha coefficient has been argued to be unrelated to a scale’s internal consistency and tends to underestimate reliability, which limits its utility despite its persistent use in psychological assessment [21]. A proposed alternative is the calculation of the glb, which is the lowest possible value that a scale’s reliability can have given the observed interitem covariance matrix [22], and another alternative is the omega total coefficient, which is a measure of reliability argued to be more robust to violations of assumptions than the alpha coefficient with less risk of over- or underestimating reliability [23]. Also, the omega coefficient is included in the present analysis as it has been argued to be a greater estimate of reliability than both the alpha coefficient and glb [24] Glb and omega total coefficients were calculated using the psych package [25] in R software [26].

We also examined concurrent validity by calculating Pearson’s product-moment correlation coefficients between the Mini-SPIN sum score, SPAI-B and SAS-A. Scores between .10 and .29 indicated a weak correlation, between .30 and .49 a moderate correlation, and .50 or greater a strong correlation [27]. All data and related metadata underlying the findings reported in a submitted manuscript are be deposited in figshare public repository: http://dx.doi.org/10.6084/m9.figshare.1472857

### 3. Results

#### 3.1. Gender and age differences

A one-way ANOVA showed that male and female participants did not significantly differ in Mini-SPIN sum scores, *F* (1, 571) = .76, *MSE* = 5.90, *p* = .38, $\eta_{p}^{2}<\;.01$. However, there was a significant main effect of age on Mini-SPIN sum scores although with a small effect size, *F* (7, 565) = 2.16, *MSE* = 5.81, *p* < .05, $\eta_{p}^{2}=\;.03$. There was no significant interaction between gender and age on Mini-SPIN scores, *F* (7, 557) = .85, *MSE* = 5.82, *p* = .55, $\eta_{p}^{2}=\;.01$ (see Table 1).

**Table 1:** Means (and standard deviations) for the Mini-SPIN.

	Community Sample (Study 1)		Clinical Sample (Study 2)		Healthy Controls (Study 2)	
*Gender*	N	M (SD)	N	M (SD)	N	M (SD)
Male	267	2.39 (2.57)	85	6.54 (2.13)	113	1.93 (1.62)
Female	306	2.22 (2.30)	62	7.11 (2.47)	94	1.67 (1.26)
*Age*						
12	10	1.30 (2.21)	3	5.00 (1.00)	3	2.33 (2.08)
13	58	2.78 (2.73)	7	8.00 (1.63)	8	2.00 (1.60)
14	138	2.75 (2.74)	41	6.68 (2.25)	44	1.70 (1.27)
15	161	2.21 (2.24)	56	6.84 (2.41)	59	1.68 (1.47)
16	122	1.91 (2.36)	40	6.73 (2.27)	47	1.96 (1.56)
17	69	1.91 (1.78)			46	1.87 (1.56)
18	15	2.67 (1.67)				

#### 3.2. Internal consistency and item analysis

Internal consistency (Cronbach’s alpha) was found to be .78, the glb was .81, and the omega total coefficient was .80 (CI 95%; .76-.84).

The average inter-item correlation was .56 (minimum = .50, maximum = .63). The mean score for item 1 of the Mini-SPIN was .52 (SD = .84), for item 2 was .57 (SD = .87) and item 3 was 1.20 (SD = 1.17).

#### 3.3. Concurrent validity: correlations with other anxiety scales

Pearson product-moment correlations were carried out for the Mini-SPIN sum score and other social anxiety measures. The correlations between the Mini-SPIN and the SPAI-B, the FNE, SAD-N and SAD-G subscales of the SAS-A and SAS-A Total score were strong (*r* = .63, 52, .51, .50, and .60 respectively).

## Study 2

### 4. Method

#### 4.1. Participants

An additional sample was comprised of 354 adolescents aged 12 to 17 years (M = 15.35, SD = 1.20), of which 168 (47.5%) were boys and 186 (52.5%) were girls. Among this sample, 147 adolescents aged 12 to 16 years (M = 15.37, SD = 1.17) had a clinical diagnosis of SAD, as measured by a diagnostic measure, of which 55 (37.4%) were boys and 92 (62.6%) were girls. Based on the DSM-5, 17 (6.8%) out of 147 met criteria for performance-only specifier. Twenty-three percent of the sample exhibited some form of comorbid disorder. In particular, 14.5% had one secondary disorder, 4.2% had two comorbid disorders, and 4.3% had 3 or more diagnoses. The most prevalent comorbid disorders were specific phobia, generalized anxiety disorder and any mood disorder. No behavioural disorder criteria were met.

In addition, this sample was comprised of 207 healthy controls (free of any diagnosis), as measured by a diagnostic measure, aged 12 to 17 years (M = 15.34, SD = 1.23); 113 were boys (54.6%) and 94 (45.4%) were girls.

#### 4.2. Measures

As in Study 1, participants were administered the SPAI-B, SAS-A and additional scales for concurrent validity.

Social Phobia Inventory (SPIN). This 17-item questionnaire measures behavioural, physiological and cognitive symptoms associated with social phobia, using a five-point likert-type scale (0 = not at all, 4 = extremely). Thus, total scores can range from 0 to 68. Although initially developed for adults, research has also demonstrated its validity and reliability in adolescent populations (for a review, please see [20]).

The Anxiety Disorders Interview Schedule for DSM-IV: Child and Parent Version (ADIS-IV-C/P; [28]). Although designed specifically to diagnose anxiety disorders, the interview also assesses mood disorders and Attention-Deficit/Hyperactivity Disorder (ADHD). Additionally, it includes screening questions for a range of other disorders (Substance Abuse, Schizophrenia, Eating Disorders, Somatoform Disorders). The social phobia section (ADIS-SP) consists of 22 dimensional ratings that evaluate social fear and avoidance using a clinical severity rating. A Clinician’s severity rating (CSR) ranging from 0 to 8 was assigned to each participant. A diagnosis of a disorder was given if the CSR was 4 or more. The ADIS-IV-C/P has moderate to strong inter-rater reliability, adequate concurrent validity, and strong retest reliability for English and Spanish-speaking populations (for a review, please see [20]). For this study, a random sample totalling 15% of child and parent interviews was audiotaped and scored by an additional interviewer who was blind to diagnosis. This interview served as the diagnostic measure.

#### 4.3. Procedure

Eight psychology students carried out the interviews within the schools’ grounds. They were previously trained in the use of diagnostic interviews, and were supervised by the study’s first author who boasts a wealth of expertise in using these instruments. The researchers conducted diagnostic interviews with the adolescents individually in a private room or office within the school. The time required for administering the assessment tool and questionnaires was approximately two hours. Adolescents with a clinical diagnosis of SAD were offered a cognitive-behavioral treatment (CBT) aimed at overcoming social fears.

The inclusion criteria were: (a) primary diagnosis of SAD, as diagnosed using the ADIS-IV-C/P; (b) subjects aged 12 to 17 years; and (c) written informed consent from both adolescent and parents. Exclusion criteria, on the other hand, were: (a) current suicidal intent or risk, and (b) a positive diagnosis of intellectual disability, psychosis, or other psychiatric conditions that would limit their ability to understand the assessment. During clinical assessment at pre-treatment, evaluators were blind to screening scores of social anxiety.

#### 4.4. Statistical analysis

ANOVA for age and gender and internal consistency analyses were conducted. We also examined concurrent validity by calculating Pearson’s product-moment correlation coefficients between the Mini-SPIN sum score, SPIN, SPAI-B and SAS-A. In addition, we examined diagnostic characteristics of the Mini-SPIN. The receiver operating characteristic (ROC) curve and area under the curve (AUC) were examined in order to determine the optimal cutpoint for diagnosing SAD in all participants, and to determine the accuracy of the social anxiety scales in comparison to the diagnostic interview. Socially anxious participants and healthy controls were compared in order to identify the screening cut-off score. The Youden Index [29] was identified as the optimal cut-off score and was used to choose the optimal scale.

### 5. Results

#### 5.1. Gender and age differences

A one-way ANOVA for the total sample showed that male and female participants did not significantly differ in Mini-SPIN scores, *F* (1, 352) = 3.15, *MSE* = 9.39, *p* = .08, $\eta_{p}^{2}=\;.01$ (see Table 1). Furthermore, there was no main effect of age on Mini-SPIN scores, *F* (5, 348) = 2.64, *MSE* = 9.54, *p* = .93, $\eta_{p}^{2}<\;.01$. There was no significant interaction between gender and age on Mini-SPIN scores, *F* (5, 342) = .69, *MSE* = 9.53, *p* = .63, $\eta_{p}^{2}=\;.01$. Among participants diagnosed with SAD, there was no significant difference in Mini-SPIN scores between male and female participants, *F* (1, 145) = 2.26, *MSE* = 11.72, *p* = .14, $\eta_{p}^{2}=\text{ 2}.02$. Also, there were no significant differences between age (12, 13, 14, 15, and 16 years old), *F* (4, 142) = .98, *MSE* = 5.24, *p* = .42, $\eta_{p}^{2}=\;.03$. Furthermore, among participants with SAD there was no interaction between age and gender on Mini-SPIN scores, *F* (4, 137) = 1.98, *MSE* = 5.05, *p* = .10, $\eta_{p}^{2}=\;.05$. Among healthy controls, there was no significant effect of gender on Mini-SPIN scores, *F* (1, 205) = 1.60, *MSE* = 2.16, *p* = .21, $\eta_{p}^{2}=\;.01$, nor was there an effect of age on Mini-SPIN scores, *F* (5, 201) = .35, *MSE* = 2.20, *p* = .88, $\eta_{p}^{2}=\;.01$, nor an interaction between gender and age, *F* (5, 195) = 1.41, *MSE* = 2.17, *p* = .22, $\eta_{p}^{2}=\;.03$. In summary, there were no statistically significant age or gender effects on Mini-SPIN scores in participants with SAD, healthy controls or the total sample.

#### 5.2. Internal consistency and item analysis

Internal consistency (Cronbach’s alpha) for all participants was .82, for participants with SAD was .55, and for healthy controls was .41. The glb for all participants was .82, for participants with SAD was .60, and for healthy controls was .44. The omega coefficient for all participants was .82 (CI 95%: .78, .85), for participants with SAD was .58 (CI 95%: .46, .70), and for healthy controls was .41 (CI 95%: .27, .54). The Mini-SPIN’s average inter-item correlation was .60 (minimum = .58, maximum = .64).

#### 5.3. Concurrent validity: correlations with other anxiety scales

Pearson product-moment correlations were carried out for the Mini-SPIN sum score and other social anxiety measures in all participants. The correlations between the Mini-SPIN and the SPIN, SPAI-B and SAS-A total scores were strong (*r* = .75, .79 and .74 respectively). The Mini-SPIN was also strongly correlated with FNE, SAD-N and SAD-G subscales of the SAS-A (*r* = .58, .70 and .68 respectively). In the clinical sample, correlations between the Mini-SPIN and the SPIN, SPAI-B, and SAS-A Total scores were moderate (*r* = .49, .47 and .42 respectively). Correlations between the Mini-SPIN and the SAS-A subscales were moderate (*r* = .38 and .42 for SAD-N and SAD-G, respectively), except for the FNE subscale (*r* = .20). In healthy controls, correlations between the Mini-SPIN and the SPIN, SPAI-B, SAS-A total scores were moderate (*r* = .43, .50 and .41 respectively). Similarly, moderate correlations were observed between the Mini-SPIN and the FNE, SAD-N and SAD-G subscales of SAS-A (*r* = .31, .36 and .32 respectively).

#### 5.4. Diagnostic efficiency of the Mini-SPIN

**Fig 1:**
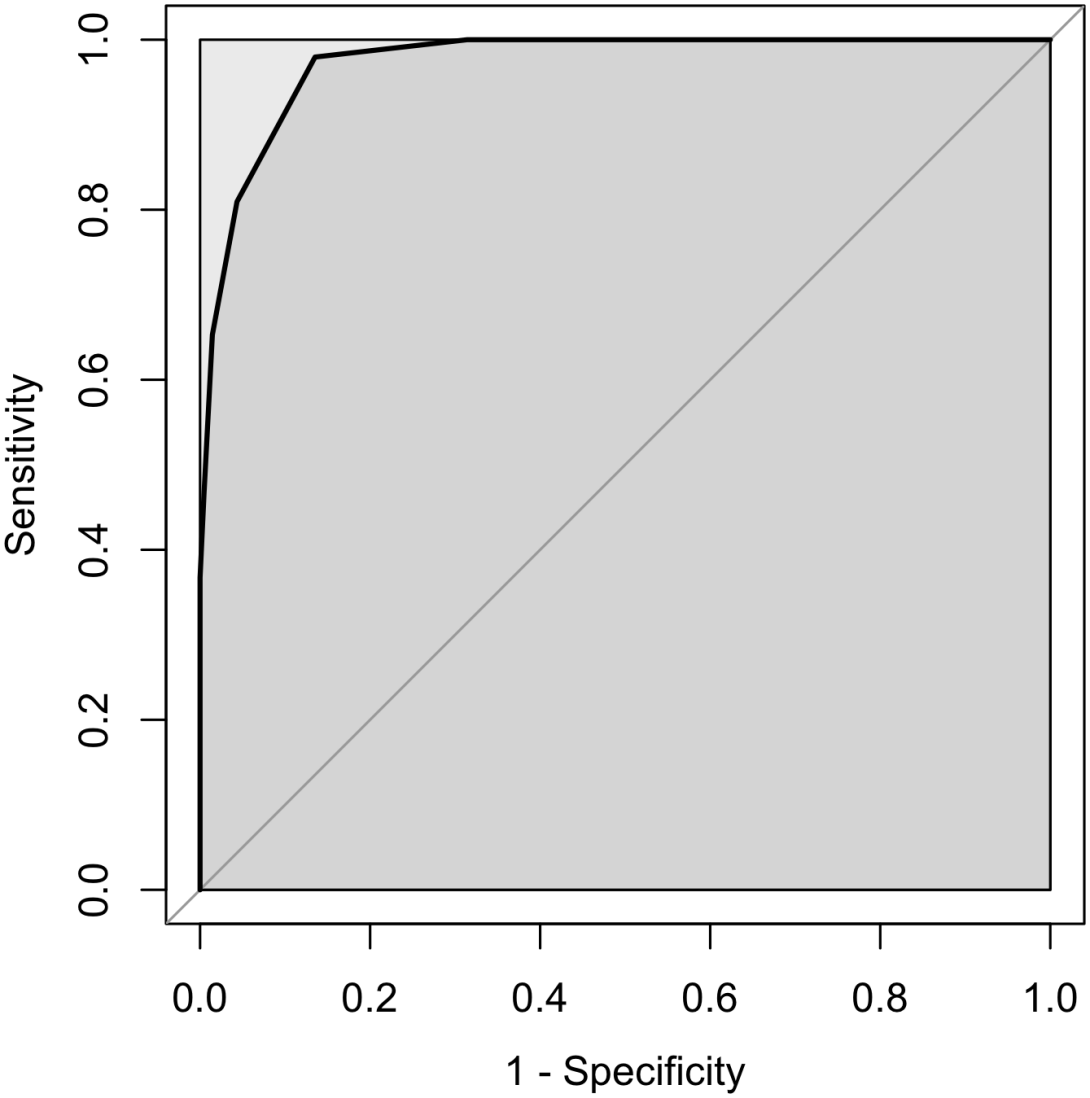
The ROC curve for participants diagnosed with SAD and control participants.

ROC analysis of adolescents with a clinical diagnosis of SAD vs. healthy controls yielded an AUC value of .97 (95% CI: .96-.99; see Fig 1). The cut-off score of ≥5 was identified as the optimal screening cut-off score where the Youden Index was .77, sensitivity was .81, specificity was .96, PPV (positive predictive value) was .93, NPV (negative predictive value) was .88, LR+ (likelihood ratio positive) = 18.62 (95% CI: 9.78–35.44), and LR- (likelihood ratio negative) = .20 (95% CI: .14-.28) (see Table 2). Out of 147 participants diagnosed with SAD, 119 (81%) reached a score of 5 or greater on the Mini-SPIN. Heterogeneity in choosing an optimal cut-off score has resulted in different cut-off scores reported across studies and populations. Which levels of sensitivity and specificity are acceptable, depends on the aim of administering the screening measure and the costs and benefits of the decision based on the screening instrument [5].

**Table 2:** Diagnostic efficiency of Mini-SPIN. The table shows sensitivity, specificity, positive predictive value (PPV), negative predictive value (NPV), likelihood ratio positive (LR+), likelihood ratio negative (LR-) and Youden’s Index (*J*).

Cutoff	Sensitivity	Specificity	PPV	NPV	LR+	LR-	*J*
≥3	147/147 = 100.0	142/207 = 68.6	147/(212) = 69.3	142/(142) = 100.0	3.18	-	.69
≥4	144/147 = 98.0	179/207 = 86.5	144/(162) = 83.7	179/(182) = 98.4	7.24	.02	.85
≥5	119/147 = 90.0	198/207 = 95.7	119/(128) = 93.0	198/(226) = 87.6	18.62	.20	.86
≥6	96/147 = 65.3	204/207 = 98.6	96/(99) = 97.0	204/(255) = 80.0	45.06	.35	.64
≥7	69/147 = 46.9	206/207 = 99.5	69/(70) = 98.6	206/(284) = 72.5	9.26	.56	.46
≥8	54/147 = 36.7	207/207 = 100.0	54/(54) = 100.0	207/(300) = 69.0	-	.63	.37

#### 5.5. Discriminative validity of the Mini-SPIN

In participants with SAD, the mean score for item 1 of the Mini-SPIN was 1.94 (SD = 1.09), for item 2 was 2.11 (SD = 1.01) and item 3 was 2.74 (SD = 1.03). In healthy controls, the mean score for item 1 of the Mini-SPIN was .45 (SD = .60), for item 2 was .51 (SD = .65) and for item 3 was .86 (SD = .89). Independent samples t-tests were carried out to examine mean score differences on each Mini-SPIN item between participants with SAD and healthy controls. Participants with SAD had significantly greater scores on item 1, *t* (352) = 16.40, *SE* = .09, *p* < .01, *d* = 1.75, item 2, *t* (352) = 18.14, *SE* = .09, *p* < .01, *d* = 1.93, and item 3, *t* (352) = 18.41, *SE* = .10, *p* < .01, *d* = 1.96. Mean Mini-SPIN sum score for participants diagnosed with SAD was 6.78 (SD = 2.29) and for healthy controls was 1.81 (SD = 1.47). A one-way ANOVA showed that the difference in mean Mini-SPIN sum scores between participants with SAD and healthy controls was greater than would be expected by chance alone, *F* (1, 352) = 617.50, *MSE* = 3.44, *p* < .01, $\eta_{p}^{2}=\;.64$.

## Discussion

The need for shortened versions of self-report measures has increased in recent years as a result of the increased importance of mental health screening by mental healthcare providers and school counsellors. The Mini-SPIN is a 3-item scale that may be particularly useful for screening purposes, although there is only limited information available on its psychometric properties for adolescents. To cover this bridge, this study reveals that the Mini-SPIN has good psychometric properties when administered on adolescents. Interestingly, the Mini-SPIN correlated stronger with the SPAI-B than with the SAS-A (particularly, for the FNE subscale) in both clinical and community samples. Whereas the SAS-A assesses cognitive and behavioural symptoms, the SPAI-B covers the triple-response-system, but assesses behavioral symptoms rather than cognitive aspects of social anxiety [13, 20]. This finding may suggest that the Mini-SPIN is more related to overall social anxiety, particularly assessing behavioral symptomatology. Surprisingly, the Mini-SPIN also correlated stronger with the SPAI-B than with the SPIN, which suggests that the Mini-SPIN is more related to the SPAI-B than the original scale. This finding merits further attention due to the scarcity of previous studies that have examined the correlations among these three scales.

It must be noted that the mean Mini-SPIN scores found in the present study reflect a continuum of severity of social anxiety symptomatology. Low Mini-SPIN scores in healthy controls demonstrates low social anxiety symptomatology, whereas elevated Mini-SPIN scores in individuals with SAD reflects high social anxiety symptomatology, with significant differences and large effect sizes between these groups. This is consistent with a dimensional conceptualization of SAD, suggesting that it exists on a continuum of severity. This notion is in line with recent studies [18, 30, 31, 32], although it differs from others [33].

Based on the AUC for the ROC analysis, the results demonstrate the discriminative capacity of the scale in differentiating adolescents with and without SAD. However, our cut-off score contrasts with previous research [10]. Mean differences in the clinical and healthy groups between Ranta *et al*. [10] and this study could account for this finding. Furthermore, consistent with studies on other social anxiety measures, there was no need for specific cut-off scores according to gender [20]. In addition, the data revealed that NPV was superior to PPV. This is consistent with research suggesting that PPV is low whereas NPV is high in populations with relatively low condition prevalence (estimate mean of 4.4% in adolescents [34]), such as SAD [35].

Based on the DSM-5, 6.8% of the clinical sample met criteria for the performance-only specifier. Limited prevalence of this subgroup has clinical implications, particularly taking into account the issues raised by authors on this subgroup [16, 18, 36, 37, 38, 39]. In particular, these studies have called attention to low prevalence of performance-only specifier and question whether it merely reflects an indicator of severity and not a different diagnostic entity.

Finally, some limitations should be noted. Internal consistency values for participants diagnosed with SAD and healthy controls were less acceptable than for the entire sample. This was due to low inter-item covariances in responses from healthy controls, and also due to low inter-item covariances in participants with SAD, although inter-item covariances were high when computed for all participants. Future studies should examine this issue. In addition, further studies should examine whether the psychometric properties of the Mini-SPIN generalize to younger populations. Similarly, cross-cultural and trans-national studies are needed even though previous research has revealed that the Mini-SPIN has similar psychometric properties across languages and cultures and it is expected that these findings may be generalizable to non-Spanish-speaking adolescents. For example, data should be replicated with other Latino groups, as people from Latin America were not included.

## Conclusions

The Mini-SPIN was designed to be reliable, valid, brief and easy to score, and administered in both clinical and non-clinical settings. Due to the simplicity and brevity of this measure, it may be particularly useful in studies of epidemiology and/or for screening purposes. The findings of this investigation are the first to complement data provided by Ranta *et al*. [10] in Finnish adolescents and indicate that the Mini-SPIN can also be recommended for screening socially anxious adolescents in a Spanish-speaking population. These data taken together suggest that the Mini-SPIN is a brief and efficient instrument for SAD in different cultures and contexts. Given that effective psychological treatments are available, we hope that the availability of useful screening measures such as the Mini-SPIN can improve the status of SAD as an under-detected condition in adolescents.
